# Spectrum of myeloid neoplasms and immune deficiency associated with germline *GATA2* mutations

**DOI:** 10.1002/cam4.384

**Published:** 2015-01-26

**Authors:** Muhammad A Mir, Samith T Kochuparambil, Roshini S Abraham, Vilmarie Rodriguez, Matthew Howard, Amy P Hsu, Amie E Jackson, Steven M Holland, Mrinal M Patnaik

**Affiliations:** 1 Penn State Milton S. Hershey Cancer InstituteHershey, Pennsylvania; 2 Division of Hematology, Blood & Marrow Transplant, Mayo ClinicRochester, Minnesota; 3 Divisions of Clinical Biochemistry and Immunology, Department of Laboratory Medicine and Pathology, Mayo ClinicRochester, Minnesota; 4 Division of Pediatric Hematology/Oncology, Mayo ClinicRochester, Minnesota; 5 Laboratory of Clinical Infectious Diseases, National Institutes of HealthBethesda, Maryland

**Keywords:** GATA2, leukemia, lymphedema, MonoMAC, viral warts

## Abstract

Guanine-adenine-thymine-adenine 2 (*GATA2*) mutated disorders include the recently described MonoMAC syndrome (Monocytopenia and *Mycobacterium avium* complex infections), DCML (dendritic cell, monocyte, and lymphocyte deficiency), familial MDS/AML (myelodysplastic syndrome/acute myeloid leukemia) (myeloid neoplasms), congenital neutropenia, congenital lymphedema (Emberger's syndrome), sensorineural deafness, viral warts, and a spectrum of aggressive infections seen across all age groups. While considerable efforts have been made to identify the mutations that characterize this disorder, pathogenesis remains a work in progress with less than 100 patients described in current literature. Varying clinical presentations offer diagnostic challenges. Allogeneic stem cell transplant remains the treatment of choice. Morbidity, mortality, and social costs due to the familial nature of the disease are considerable. We describe our experience with the disorder in three affected families and a comprehensive review of current literature.

## Introduction

The guanine-adenine-thymine-adenine (*GATA*) family is comprised of six zinc-finger transcription factors that recognize approximately 7 million *GATA* motifs in the human genome 1,2. *GATA1* is instrumental in development of erythrocytes, mast cells, eosinophils, and megakaryocytes 3–8 and is implicated in Down syndrome-related acute megakaryocytic leukemia and transient myeloproliferative disorder 9,10. *GATA1* is also associated with X-linked thrombocytopenia and dyserythropoeitic anemia (Diamond–Blackfan anemia) 75–77. *GATA2*, located on 3q21 11 is pivotal in proliferation of hematopoietic stem cells (HSC) and mutations were first described in aplastic anemia 12–15. Pedigree studies have initially recognized two mutations in *GATA2* in familial AML, p.T354M, and p.T355del, both in the second zinc finger (ZF-2) of *GATA2* 16–18, while two other mutations; p.R308P and p.A350-N351ins8 are associated with de novo AML 78. During erythropoiesis; GATA switching results in displacement of GATA2 by GATA1 from chromatin causing inhibition of GATA2, promoting downstream erythroid differentiation 5,19. In contrast, *GATA2* overexpression induces megakaryocytic differentiation in cell lines 20. *GATA2* exerts an inhibiting influence on the *PU.1* gene which is essential for monocytic, granulocytic, and lymphoid differentiation 21. In contrast to *RUNX1*, which is essential for generation of HSC, *GATA2* appears to be essential for HSC generation and subsequent survival 22. Other *GATA* genes perform a diverse array of functions. *GATA3* promotes T-cell lymphopoiesis 23–28 but deficiency has been associated only with hypoparathyroidism, deafness, and renal disease. *GATA4* has recently been implicated in childhood onset diabetes 29. *GATA 5* CpG island hypermethylation in renal carcinoma appear to identify aggressive phenotypes with poor outcomes 30. In animal models, *GATA6* has been shown to orchestrate cardiac muscle hypertrophy in response to pressure stress and increase hepcidin expression in inflammatory states 31,32. In summary, GATA factors 1–3 appear to be involved in hematopoiesis, while GATA 4–6 appear to be more important for cardiac development and function 33 although expression has been demonstrated in other endodermal and mesodermal organs such as lung, liver and gonads and gut 34.

The study of germline mutations such as *GATA2* provides profound insights into leukemogenesis, immune dysfunction and cross-talk of seemingly diverse genetic pathways such as *CEBPA, PU.1* 35–38, and *RUNX1* 39–43. The clinical phenotype of germline *GATA2* mutations include, but is not limited to, spectrum of immune deficits such as MonoMAC syndrome 44–46, dendritic cell, monocyte and lymphoid deficiency (DCML) 47, familial MDS (myelodysplastic syndrome)/AML (acute myeloid leukemia), and Emberger's syndrome 48. Of note, sporadic mutations in *GATA2* are described and may have no familial implications as described below. Our focus in this article is the haploinsufficiency induced by spontaneous germline mutations in *GATA2* resulting in an autosomal dominant inheritance of diverse phenotypes 44,46,49. The differential diagnosis of *GATA-2* deficiency includes other related disorders with overlapping features and are summarized in Table1.

**Table 1:** Mutations/disorders in differential diagnosis of *GATA2* deficiency

Familial MDS/AML 67	Warts/HPV infections 51	Mycobacterial infections 68–70	Congenital lymphedema 71–73,75,76	Pulmonary alveolar proteinosis 77
*TERT/TERC* *CEBPA* *RUNX1*	DOCK 8 CXCR4 HIV/CD4↓ TMC6/8 SPINK5/LEKT1 STK4/MST1	*IFNGR1* *IFNGR2* *IL12RB1* *STAT1* (loss of function; AR and AD) *IRF8* *CYBB* (macrophage-specific mutation) *TYK2* *ISG15* *IKKG* (NEMO)	*FLT4* *GJC2* *FOXC2* *SOX18* *CCBE1* *PTPN14*	Anti-GM-CSF Ab *CSF2RB*

MDS, myelodysplastic syndrome; AML, acute myeloid leukemia; HPV, human papillomavirus.

## Case Series

### Family 1

The proband is a 38-year-old Caucasian male, who presented with progressive dyspnea and fatigue of 3 months duration and was found to have pancytopenia. A bone marrow biopsy revealed hypocellular marrow but demonstrated acute myeloid leukemia (AML) with the following cytogenetic abnormalities: t (1; 21) (q10; q10) [9]/+8[4]/46XY [7]. He received standard induction chemotherapy with idarubicin and cytarabine, and his course was complicated by an orbital fungal infection with *Absidia lithemia*, medically and surgically managed, following which he underwent a reduced-intensity conditioning-matched unrelated donor allogeneic hematopoietic stem cell transplant (MUD-Allo-HCT). Posttransplant course was complicated by severe refractory immune-mediated thrombocytopenia requiring a splenectomy and an orbital relapse of AML. Due to history of multiple family members being affected (Fig.2) by AML and extra genital warts (sister, son, and daughter), congenital lymphedema (son), and cytopenias (sister) a work-up for familial bone marrow failure syndromes was carried out. *GATA2* mutation analysis performed at the National Institutes of Health (NIH) confirmed the presence of a missense mutation (1339A>C, p S447R) in the patient, a female sibling, a son, and a daughter. The female sibling with MDS and viral warts also underwent MUD-Allo-HSCT (hematopoietic stem cell transplant) and remains symptom-free 14 months posttransplant. The probands son and daughter also underwent MUD allogeneic HSCT and are doing very well more than 12 months posttransplant. Patient characteristics and outcomes are shown in Table2.

**Table 2:** Clinical characteristics and outcomes of patients with GATA2 mutations that underwent allogeneic stem cell transplantation

No.	Diagnosis Age/Sex	Cytogenetics/GATA2 mutation	Associated features	Prior Rx	HCT-CI	CMV	Regimen	ABO/HLA	GVHD Prop	aGVHD Status	Complications	Day 30 Chimerism /Marrow	Day 100 Chimerism /Marrow	Last follow up (Days)
1	AML 38/M	46,XY, t(1;21) [9] +8[4]46,XY [7] S447R	NK/B-cell def Viral warts Hydrocele	Idarubicin Cytarabine	0	D+ R+	Fludarabine TBI (200)	ABO− mismatch HLA 9/10	Tacrolimus MTX	GI Grade 1	E.Faecalis CMV Anti-platelet antibodies/Thrombocytopenia	100% Donor 30% Hypo- Cellular	100% Donor 30% Hypo- Cellular	356 (extra medullary relapse day 307)
2	MDS 35/F	S447R	NK/B-cell def Viral warts Infertility	None	0	D− R+	Busulfan Cytoxan	ABO− match HLA 10/10	Tacrolimus MTX	Skin Grade 1	CMV	100% Donor 30% Hypocellular	100% Donor 60% Hypocellular	208
3	MDS 10/F	S447R	NK/B-cell def Viral warts	None	0	D− R+	Cytoxan, TBI Alemtuzumab	ABO− match HLA 9/10	Tacrolimus MTX	Skin Grade 1	C.difficile	100% Donor 30% Hypo- Cellular	100% Donor 20% Hypo- Cellular	247
4	Chronic Neutropenia 7/M	S447R	NK/B cell def Viral warts Emberger's syndrome Pulmonary stenosis	None	0	D− R+	Cytoxan, TBI Alemtuzumab	ABO− match HLA 10/10	Tacrolimus	–	–	Pending 30% Hypo cellular	NA	31

MDS, myelodysplastic syndrome; AML, acute myeloid leukemia.

**Figure 1:**
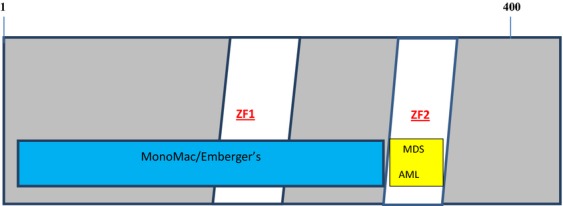
Genotype/phenotype relations of GATA2 mutations.

### Family 2

Family 2 was discovered by the birth of a newborn with dysmorphic features (head size larger than stomach) resulting in a cytogenetic examination in infancy with identification of deletion 3q13.2-q21.3, which includes the *GATA2* gene. The child exhibited monocytopenia without lymphopenia or neutropenia. Dendritic cell activity was not assessed for. The parents were tested and did not have the same gene defect. She is being followed with monthly blood tests and has not demonstrated any systemic infections or signs of MDS/AML although cognitive development appears to be delayed.

**Figure 2:**
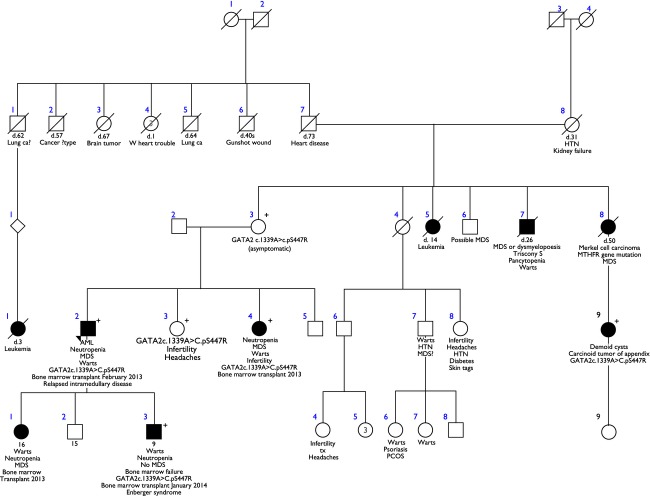
Family Tree of Proband 1 with the GATA2 c.1339A>C.pS447R mutation.

**Figure 3:**
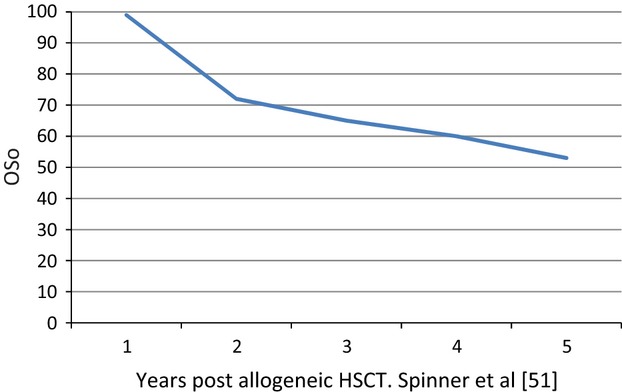
Years post allogeneic HSCT for GATA2 mutation Spinner et al. [53]HSCT, 5 hematopoietic stem cell transplant.

### Family 3

In Family 3, the proband is a Caucasian female who presented at age 17 years with abdominal pain, hemoptysis, and mild pancytopenia. A CT scan revealed mild diffuse thoracic and abdominal lymphadenopathy. A detailed evaluation found acute Epstein–Barr virus (EBV) infection. A bone marrow biopsy was mildly hypocellular with mild erythroid hypoplasia and megakaryocytic hyperplasia with atypia. The cytogenetics were 46, XX 20. She had a history of recurrent episodes of hidradenitis suppurativa, skin abscesses, folliculitis, otitis media, and throat infections. Several years later, she was initiated on therapy with pegylated G-CSF. In spite of this, otitis media and abscesses continued. Two years later, she presented with a hypercatabolic state, with progressive hepato-splenomegaly and constitutional features. A bone marrow biopsy demonstrated progressive megakaryocytic atypia. While cytogenetics were once again normal, however, a MDS-fluorescence in situ hybridization (FISH) panel identified a deletion of -3q21 in 99% of analyzed nuclei. A phytohemagglutinin-stimulated karyotyping of peripheral blood lymphocytes also demonstrated the -3q21 (RPN1 deletion) in 99% of analyzed nuclei. *GATA2* is located within this region, Expression studies confirmed *GATA2* haploinsufficiency. She is awaiting a donor for a MUD-HSCT.

## Discussion

### MonoMAC syndrome and DCML deficiency

The terms MonoMAC and DCML are synonymous, in terms of the genetic etiology, and refer to a primary immunodeficiency with predisposition to MDS/AML. MonoMAC refers to a recently described syndrome of *MONO*cytopenia and *M*ycobacterium *A*vium *C*omplex infections characterized by germline *GATA2* mutations 44,46. DCML, also caused by germline *GATA2* mutations refers specifically to the cytopenias frequently seen in most patients—DCML deficiency (both B and NK cell) 50. Two independent groups studied 24 individuals with these syndromes and reported similar mutations noted above in familial syndromes (T354M and T355 del). The scope of immune deficiency in this group is vast and not limited to mycobacterial infections. Opportunistic viral (disseminated human papillomavirus [HPV] and HPV-associated squamous cell carcinoma) 51, parasitic and fungal infections, as well as pulmonary alveolar proteinosis (*GATA2* is known to influence the phagocytic activity of pulmonary alveolar macrophages) can be seen 52. A majority of patients with *GATA2* mutations eventually show deficiency of B lymphocytes, NK cells, CD4 lymphocytes, and monocytes 53.

#### Emberger's syndrome

Emberger's Syndrome is primary lymphedema with cutaneous warts, deafness, and a propensity to develop MDS/AML. Intact *GATA2* function is required for proper lymphatic vascular development during embryogenesis in mice 54. Ostergaard and colleagues identified eight mutations in *GATA2* in three patients with this syndrome by whole-exome sequencing identifying this mutation as the only common denominator between the group 48. At least 1 other patient with propensity to varicella zoster and salmonella infections has been reported 48. Complications secondary to prolonged lymphedema such as secondary cellulitis and deep vein thrombosis (DVT) are frequent 53. Null mutations in *GATA2* appear to be associated with severe viral infections and lymphedema 52.

### Familial MDS/AML

*GATA2* overexpression has been documented in one-third to one half of nonfamilial AML and correlates with a poor prognosis with shorter overall and event-free survival when treated with standard chemotherapy 55,56. Of the original four families with *GATA2* mutations, described by Hahn et al. with MDS/AML, three had the T354M mutation, and one had deletion T355. Both mutations occurred in the second zinc finger (ZF) of GATA2 (Fig 1). In the T354 mutation families, all members had the mutation but not all had developed hematological disease at least by the time of reporting 16. Bone marrow biopsies are typically hypocellular in contrast to the common MDS marrow picture, with abundant atypical megakaryocytes in >90% patients 53. Some patients have also fulfilled the diagnostic criteria for CMML (chronic myelomonocytic leukemia) and LGL (large granular lymphocytic leukemia) suggesting overlap syndromes 53. Other acquired mutations such as *ASXL1* may herald the development of AML 57. Increased levels of *FLT3* ligand have also been reported to be associated with clinical progression 58.

#### Chronic myeloid leukemia

A novel *GATA2* mutation *L359V* has been found in nearly 10% of patients with accelerated or blast phase CML, but not CLL or ALL 59,60. This is thought to be mediated through *PU.1* inhibition. It is interesting to note that GATA2 overexpression or the *L359V gain-of-function* mutation have been associated with AML and CML, respectively; whereas *loss-of-function* mutation of GATA2 such as *T354M* have been linked to MDS. L359 and T354 located in the same region on the second zinc finger of GATA2 thus highlighting the vital role GATA2 plays in hemostasis of myeloid precursors.

### Aplastic anemia

Expression of GATA-2 mRNA in purified CD34-positive cells was significantly decreased in aplastic anemia compared with normal subjects when examined by immunocytochemical analysis 61. The changes extend further to stromal cells, with lower expression of GATA2 in patients with aplastic anemia when compared to controls by RT-PCR-ELISA 62. *GATA-2* is instrumental in both hematopoiesis and adipogenesis. Overexpression of peroxisome proliferator-activated receptor-gamma (PPAR-*γ*, an adipogenic factor) and underexpression of GATA2 by mesenchymal stem cells may explain fatty marrow replacement in AA patients 63.

### Pediatric neutropenia

A high frequency of *GATA2* mutations has been reported in pediatric patients with mild chronic neutropenia 64. Analysis of French Neutropenia registry data revealed chronic familial neutropenia in seven families predisposing to MDS/AML associated with *GATA2* mutations that included a complete deletion of *GATA2* locus as well as additional mutations (p.R396Q, R204X, R330X, E224X, A372T, and M388V) 64.

### Pulmonary disease

Ventilation-diffusion defects can be demonstrated in about two-thirds of *GATA2*-deficient patients while pulmonary hypertension (PAH) and pulmonary alveolar proteinosis (PAP) are some of the rare manifestations occurring in <20% in one series 53. PAP in GATA2 deficiency is not due to GM-CSF-(Granulocyte Monocyte- Colony Stimulating Factor) autoantibodies and is refractory to GM-CSF inhalational and subcutaneous therapy 53.

## Treatment

### Immune deficiency

Allogeneic HSCT remains the main therapy for *GATA2*-deficient patients with immunodeficiency. Timing of HSCT for immune deficiency alone is less well defined as compared to MDS/AML and should focus on risk-benefit ratios for the individual and the family. The incidence of HPV, mycobacterial, and fungal infections decreases considerably after successful allogeneic HSCT 53,65. Notably, it may take more than 3.5 years for reversal of phenotype and full immune reconstitution of B, NK, and monocyte populations 66. This may be especially problematic with delayed engraftment typical of umbilical cord grafts. Both PAP and PAH also respond well to HSCT and repeated lung infections or declining lung function should be considered an indication in clinical context 53. Earlier transplantation, before organ dysfunction ensues, results in less morbidity and mortality.

### MDS/AML

Allogeneic HCT remains the only treatment with favorable responses in *GATA2*-mutated MDS/AML (Fig.3). In the NIH experience, 21 patients were transplanted for either hematological (MDS/AML) or immunological indications (age 15–49 years) with good responses (Fig.1). Of note, half the patients who were not transplanted passed away by age 40 51. A similar NIH experience further outlined use of conditioning regimens for nonmyeloablative allogeneic HCT 66. Donors included fully matched related and unrelated donors (conditioning-fludarabine + total body radiation 200 cGy) and alternative sources such as umbilical cord blood and haploidentical bone marrow (fludarabine + cyclophosphamide and total body irradiation 200 cGy, with posttransplant cyclophosphamide for T-cell replete grafts). Busulfan was later added for a more robust eradication of the *GATA2* clone. Azithromycin was started before and continued for 1 year posttransplant due to increased propensity to nontuberculous mycobacterial (NTM) infections, in addition to standard prophylaxis. No NTM infections during or after transplant were reported using prophylaxis. Overall survival was 57% at 36 months. Our patient characteristics and outcomes are shown in Table2. Tacrolimus was used for graft versus host disease prophylaxis. All four patients have engrafted with 100% donor chimerisms (CD3 and CD33 fractions). One patient had CMV-Cytomegalovirus reactivation and refractory thrombocytopenia which failed to improve despite splenectomy. Three developed acute GVHD and one had chronic GVHD involving esophagus with dysphagia and strictures that improved with steroids.

Two clinical trials are currently recruiting patients for myeloablative and reduced-intensity conditioning allogeneic HSCT for *GATA2* mutations and enrollment is encouraged whenever feasible (NCT01861106 and NCT00923364 at www.clinicaltrails.gov).

### Genetic counseling

Early genetic diagnosis and screening is paramount 53. Patients and families should be seen in conjunction with a geneticist. Suggested screening categories are listed in Table3. Variable phenotypes resulting from different GATA2 mutations are listed in Table4.

**Table 3:** Suggested screening categories for *GATA2* mutation

Pediatric neutropenia
Monocytopenia
B-cell or NK cell cytopenia
Dendritic cell deficiency
MDS with hypocellular bone marrow
Familial MDS/AML
PAP in absence of anti-GM-CSF autoantibodies
Recurrent extra genital HPV warts or severe refractory genital HPV
Severe viral infection (HSV, EBV)
Lymphedema, often later onset
Sensorineural deafness with immunodeficiency
Disseminated nontuberculous mycobacteria (NTM)
Disseminated or severe fungal infections
Pulmonary NTM without bronchiectasis

Modified from Spinner et al. 53. MDS, myelodysplastic syndrome; AML, acute myeloid leukemia; HPV, human papillomavirus; PAP, pulmonary alveolar proteinosis; EBV, Epstein–Barr virus.

**Table 4:** Mutations of *GATA2* resulting in variable phenotypes (Fig.3) 78

Mutation type	AA location	Phenotype
Nonsense	337(ZF1)	Emberger syndrome
Missense	254	MonoMAC/DCML
	354(ZF2)	Familial MDS/AML, MonoMAC/DCML
	361(ZF2)	MonoMAC/DCML
	371(ZF2)	MonoMAC/DCML
	373(ZF2)	Emberger syndrome
	396(ZF2)	MonoMAC/DCML
	398(ZF2)	MonoMAC/DCML
Frameshift	1	MonoMAC/DCML
	78	Emberger syndrome
	81	MonoMAC/DCML
	105	Emberger syndrome
	194	Emberger syndrome
	200	MonoMAC/DCML
	259	MonoMAC/DCML
	317(ZF1)	MonoMAC/DCML
	341(ZF1)	Emberger syndrome
In-frame insertion or deletion	355(ZF2)	Familial MDS/AML
	361(ZF2)	MonoMAC/DCML, Emberger syndrome
Large deletion	340–381(ZF1 & 2)	MonoMAC/DCML

MDS, myelodysplastic syndrome; AML, acute myeloid leukemia; DCML, dendritic cell, monocyte, and lymphocyte.

## Conflicts of Interest

None declared.
